# Conducting COVID-19-Related Research in Jordan: Are We Ready?

**DOI:** 10.1017/dmp.2020.437

**Published:** 2020-11-05

**Authors:** Balqis Ikhmais, Alaa M. Hammad, Walid Al-Qerem, Osama H. Abusara, Jonathan Ling

**Affiliations:** 1 Department of Pharmacy, Faculty of Pharmacy, Al-Zaytoonah University of Jordan, Amman, Jordan; 2 Faculty of Health Sciences and Wellbeing, University of Sunderland, Chester Road, Sunderland, United Kingdom

**Keywords:** COVID 19, research evaluation, virology research, readiness of research

## Abstract

The coronavirus disease-2019 (COVID-19) pandemic is a public health emergency of international concern. This pandemic poses a challenge to research and scientific community. In this study, we developed and tested content reliability and content validity of a questionnaire designed for evaluating the readiness and willingness of researchers to participate in virology research in Jordan. The survey was hosted on an online platform, and the link was emailed. A total of 332 participants from universities across Jordan completed the survey. For factor analysis, Kaiser-Meyer-Olkin value (KMO) and Bartlett’s Test of Sphericity were conducted. Furthermore, exploratory factor analysis (EFA) with parallel analysis and scree plots were conducted to evaluate the most suitable model for the data. The result of the EFA suggested a 5-factor model would fit the survey. Data showed that the lowest means were for researchers’ readiness to conduct virology research and readiness for virology research with means of 2.07 and 2.95, respectively. Moreover, years of experience and speciality had a significant effect on the readiness and willingness of virology research in Jordan. In conclusion, readiness for research and researchers should be addressed and authorities should pay attention to these shortcomings in virology research.

In recent years, virus-caused infections have had considerable prominence in public health.^1^ Numerous new viruses have been identified in the past 3 decades, including human immunodeficiency viruses (HIV), hepatitis B and C, severe acute respiratory syndrome (SARS) and avian influenza.^2^ These isolated viruses have played a significant role in developing a new model of public health perceptions. Similarly, the social and economic structure of global communities has been affected by the emerging viruses.^3^

A previous editorial report emphasized on the importance of research, especially virology research.^4^ The growing significance of virology is directly related to the fact that we know more and more viruses, better understand their ties to certain diseases, and that certain viral infections are looked at in different ways by epidemiology: suddenly we identify viruses where we have not seen them before.^5^ Case in point: diseases of the Zika virus or the spread of the Chikungunya virus, which has conquered many new areas over the past few years.^6^ At the same time as the viruses were multiplying, however, our diagnostic capabilities were expanding tremendously and groundbreaking progress has been made in viral therapy.^7^ For example, modern antiviral medication therapies for hepatitis C have all but revolutionized conventional therapies that have been fraught with side effects.^8^ Virus infections will continue to spread with the on-going globalization. For all intents and purposes that will inevitably extend to the entire virology discipline, the treatment of triggers and diagnostics will become more relevant.^9^

In December 2019, a pneumonia outbreak related to a new coronavirus, termed severe acute respiratory syndrome coronavirus 2 (SARS-CoV-2) was first discovered in Wuhan, China.^10^ Thenceforth, infection spread across China and become globalized.^11-13^ The disease caused by the novel coronavirus was identified by World Health Organization (WHO) on February 12, 2020 as coronavirus disease 2019 (COVID-19).^14^ By the end of September 2020, more than 33 million confirmed cases were reported and more than 1 million deaths had been attributed to coronavirus infection.^15^ Furthermore, COVID-19 has had a devastating impact on economies through numerous routes, including health, transportation, agricultural, and tourism.^16,17^ Correspondingly, trade with other countries may also be affected, as modern economies’ interconnectivity means that an outbreak may also affect international supply chains.^17^ Therefore, to respond efficiently to the COVID-19 outbreak, quick identification of cases and contacts, suitable clinical management and infection control, and employment of community mitigation efforts are essential.^18^

Importantly, there is an urgent need to enhance the efficiency of the research process involving the study of pathogenesis, mechanisms of spreading and developing treatment and vaccines for COVID-19. To do so, all the research elements should be ready and effective. In this study, we analyzed the perceptions of Jordanian academics and researchers in medicine and associated disciplines, including microbiology and pharmacology related to virology to develop suggestions to prepare virology research for future outbreaks. As the demand for virology research increases globally during the COVID-19 pandemic, we also measured the readiness and effectiveness of virology research in Jordan. Therefore, this study aims to develop a valid and reliable tool to evaluate the readiness and willingness of researchers to conduct virology research. Furthermore, we intend to highlight perceived obstacles and difficulties that may have the potential to delay the progression of virology research.

## Methods

### Design and Ethics

This was a cross-sectional survey directed toward academics and researchers from different medical/biological faculties in Jordan. This survey was developed to study 2 main objectives. The study was approved by Al-Zaytoonah University of Jordan Research Ethics Committee (Ref. 8/18/2018-2019). A panel of 4 academic pharmacists, who hold PhDs with varying expertise in various disciplines, including survey design, English and pharmacy education, performed face and content validations. The survey was pilot-tested on 10 faculty members to assess their comprehension of the survey and the time taken to complete it. The results obtained from these faculty members were excluded from the study. Following the pilot study, suggestions were given on ways to improve the technical aspects of the survey, such as the number of the questions or the order of the answers, which was amended accordingly.

### Survey

A literature review was conducted to prepare questions. The guidelines on establishing a virology research laboratory in developing countries, WHO Laboratory Biosafety Manual,^3,19^ and the perception of academics and researchers working in health-related fields were taken into consideration. The survey consisted of 2 sections. The first section consisted of 9 multiple-choice questions that asked for anonymous demographic information about the respondent. The second section consisted of 37 questions to measure respondents’ views of different aspects relating to the readiness and effectiveness of virology research in Jordan, including COVID-19. Participants were asked to rate their opinions on a 5-point Likert scale from 1 (strongly disagree) to 5 (strongly agree) (Appendix 1). The survey took approximately 15-20 min to complete.

### Sampling and Sample Recruitment Process

The study population included academic and research staff from medical and science faculties (health-related faculties) at public and private institutions. The institutions included universities and research centers across Jordan. Examples of health-related programs were medicine, veterinary science, doctorate in pharmacy, pharmacy, nursing, medical analysis, genetic engineering, chemistry, and biology.

The heterogeneity of the population was intentionally high because the purpose of this study was to develop a generalized instrument that could be used to evaluate the readiness extent of human virology research in different countries across the globe. The sampling strategy was purposive, and the survey was administered to participants working in various universities across Jordan. The number of participants used to validate this instrument was calculated based on a question to participant ratio of 1:5 as previously described.^20^ Moreover, our study population included all researchers who were interested in medical research in different disciplines. The total number of study population was 2000. Therefore, using sample size calculation (Kish formula) for a finite population, the required sample size to obtain 5% margin of error is 323. Importantly, the sample size in this study (332) satisfied these requirements.

The survey was distributed to academic and research staff from medical and science faculties (health-related faculties) at 21 public and private universities across Jordan. The survey and the participant information sheet were hosted on an online platform (google form), and the link was emailed. No financial incentives were offered, and reminder emails were sent out to all faculties 2 wk after the initial email. The questionnaire was distributed and data collected between April 2020 and May 2020.

## Data Analysis

Categorical variables were expressed as frequencies and percentages while continuous variables were expressed as means and standard deviations (SDs). The survey questions were treated as ordinals. The Kaiser-Meyer-Olkin value (KMO) and Bartlett’s Test of Sphericity were conducted to evaluate the suitability of the data for factor analysis. Parallel analysis and scree plots were evaluated to determine the suitable number of factors to be included in the model. Exploratory factor analysis (EFA) was conducted using principal-components analysis (PCA) to evaluate the most suitable model for the data. The correlation matrix indicated that the factors generated were highly correlated (r = −0.49); thus, oblimin rotation was used to generate a pattern matrix. Communalities were examined, and items that were below 0.3 were excluded. In addition, items that had loadings of 0.4 or greater in more than 1 factor or did not have a loading of 0.4 or greater in any factor were excluded. The factor correlation matrix was evaluated to determine discriminant validity. Cronbach’s alpha was evaluated to assess the internal consistency of each factor. The ceiling and floor effects were evaluated by calculating the frequency of participants that had the maximum or minimum possible scores; the percentage of participants who have these scores should not exceed 15.^21^

Confirmatory factor analysis (CFA) was conducted to re-evaluate the suitability of the data to the suggested model using the Bengt Muthén method, maximum likelihood extraction method was used. Goodness of fit of model was evaluated by examining comparative fit index (CFI), incremental fit index (IFI), root mean square error of approximation (RMSEA), and minimum discrepancy per degree of freedom (CMIN/DF), the values of IFI and CFI of 0.8 or greater, RMSEA of 0.1 or less.^22^ Alternative ways of assessing model fit where CMIN/DF is less than 3 are considered acceptable.^23,24^

Dummy variables were created for questions that allowed multiple options. We used Mann Whitney U test, t-test, analysis of variance (ANOVA), and Kruskal-Wallis 1-way analysis of variance were applied to evaluate differences in factor scores between each subgroup. Several analysis of covariance (ANCOVA) models were conducted to evaluate the association of the demographic variables with the score of each factor. Repeated measures ANOVA was conducted to evaluate the differences between the means of the scores for each factor. Finally, all data analysis was conducted using AMOS version 22 and SPSS version 25.

## Results

A total of 332 participants (16.6% response rate, number of respondents divided on the total number of study population) from different faculties completed the survey. Approximately half of the correspondents were male (55.4%). The majority of respondents had a doctorate degree (84.0%). Almost all of the correspondents were academic researchers (93.4 %). Of the entire study sample, 60.5% worked in a public university. When asking about research interests, 62.0% had research interest in medical field. More than third of the participants (35.2%) had research experience of 5 y or less. Full sociodemographic characteristics of participants are illustrated in Table 1.

**Table 1:** Sociodemographic characteristics of participants

Variables		Frequency and Percentage (%)
Gender	Male	184 (55.4%)
	Female	148 (44.6%)
Education level	Bachelor’s degree	7 (2.1%)
	Master’s degree	46 (13.9%)
	Doctorate degree	279 (84.0%)
Your position	Administrative	32 (9.6%)
	Academic-researcher	310 (93.4%)
	Research assistant	27 (8.1%)
Institution type	Public university	201 (60.5%)
	Private university	131 (39.5%)
Research interest?	Medicinal chemistry	127 (38.3%)
	Biology	130 (39.2%)
	Medical fields	206 (62.0%)
Years of research experience	≤5	117 (35.2%)
	6-10	83 (25.0%)
	11-15	57 (17.2%)
	16-20	26 (7.8%)
	>20	49 (14.8%)

**Figure 1:**
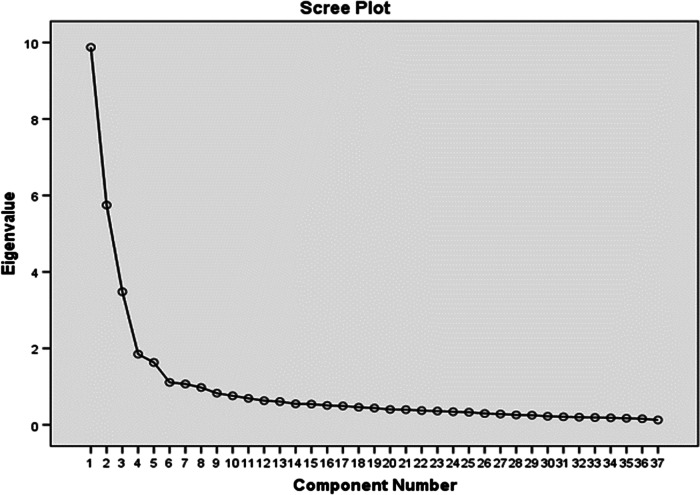
Screen plot

The Kaiser-Meyer-Olkin test result was 0.9 and Barlett’s test of sphericity was significant, *χ* ^*2*^(666) = 7748.47, *P* < 0.01, indicating the suitability of the data for factor analysis. The communality of the question “I have enough time to conduct scientific research” was 0.18 and, thus, was excluded from the final model. The scree plot (Figure 1) and parallel analysis suggested a 5-factor model.

These factors were the expected factors when formulating the questionnaire, which included readiness of virology research (RVR), willingness for conducting virology research (WCVR), respondents’ opinions regarding virology research components (ROVRC), readiness of scientific research (RSR), and readiness of the researcher (RR). As Table 2 shows, in the retained items in the final model the lowest communality was 0.43 for the item “In my opinion, there is a high need to increase the number of microbiologists (including virologists) in my institution to enable the conduction of a research in the field of human viral infectious diseases (including COVID-19)” and the lowest factor loading was 0.45 for “My institution has clear regulations that allow joint research collaborations with various institutions (including the healthcare sector).”

**Table 2:** Factor loadings, communalities, reliability, and factor means ± SD

	Factor Loadings	Communalities Min-Max	Cronbach’s Alpha	Corrected Item-Total Correlation	Mean ± SD
RVR Q15, Q25, Q26, Q28, Q29, Q31, Q32, Q33, Q34	0.47-0.81	0.52-0.68	0.91	0.89 −0.90	2.95 ± 0.74
WCVR Q6, Q7, Q8, Q9, Q10, Q11, Q12	0.54-0.91	0.51-0.85	0.92	0.90-0.93	3.35 ± 1.01
ROVRC Q18, Q19, Q21, Q27, Q30, Q36, Q37	0.80-0.58	0.49-0.65	0.83	0.46-0.68	4.21 ± 0.58
RSR Q13, Q14, Q16, Q17, Q20, Q22, Q23, Q24, Q35	0.44-0.81	0.43-0.65	0.90	0.54-0.73	3.42 ± 0.77
RR Q1, Q2, Q3, Q4, Q5	0.68-0.78	0.61-0.70	0.88	0.67-0.75	2.07 ± 0.91

RR, readiness of the researcher; RSR, readiness of scientific research; ROVRC, respondents’ opinions regarding virology research components; RVR, readiness of virology research; SD, standard deviation; WCVR, willingness for conducting virology research.

**Figure 2:**
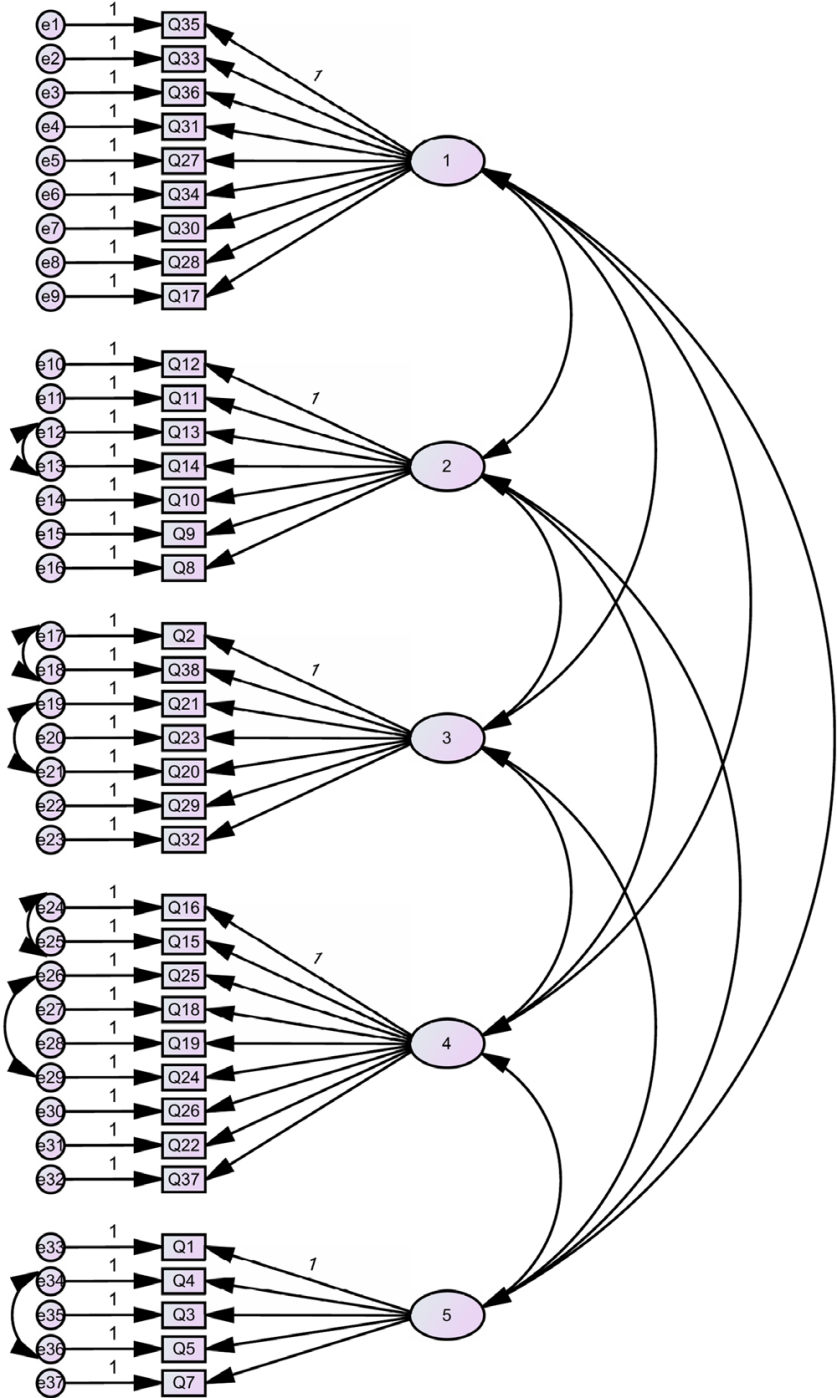
Confirmatory factor analysis

Cronbach’s alpha values were all above 0.8, and deleting any item would not improve the reliability indicating good internal consistency. Correlations between the 5 factors was evaluated using Pearson’s correlations, and the highest correlation was 0.49, indicating acceptable discriminant validity. CFA confirmed the 5-factor model with acceptable model fit indicators (CFI = 0.90, IFI = 0.89, RMSEA = 0.06, and CMIN/DF = 2.2) (Figure 2). Ceiling and floor effects were not present as the percentages of respondents that had the maximum or minimal possible scores were less than 15%.

As Table 3 shows, several variables were associated with at least 1 factor score including education level, institution position, type of institution, research interest, and years of experience. Position in the institution was significantly associated with RVR, RSR, and RR as the means of RVR, RSR, and RR were significantly lower in “academic researchers” when compared with “non-academic researcher” and significantly higher in “research assistant” when compared with “not research assistant.” RR mean was significantly higher for bachelor’s degree when compared with other education levels. RVR was significantly higher for public university than private. “Medicinal chemistry research interest” had significantly higher means in WCVR and RR when compared with “no medicinal chemistry research interest”, RR mean was also significantly higher in “having biology research interest” when compared with “not having biology research interest.” The highest mean in RR was in 11-15 y of experience group when compared with other years of experience groups, while in RSR the highest mean was in >20 y of experience group.

**Table 3:** Association between different sample characteristics and factors’ means

Independent Variables			RVR (Mean ± SD)	WCVR (Mean ± SD)	ROVRC (Mean ± SD)	RSR (Mean ± SD)	RR (Mean ± SD)
Gender	Male		2.99 (±0.79)	3.36 (±1.04)	4.20 (±0.62)	3.47 (±0.76)	2.15 (±0.93)
	Female		2.90 (±0.67)	3.35 (±0.99)	4.22 (±0.52)	3.36 (±0.78)	1.97 (±0.87)
Education level	Bachelor’s degree		3.32 (±1.0)	3.82 (±0.86)	4.14 (±0.73)	3.95 (±0.59)	3.20 (±0.86)**
	Master’s degree		3.0 (±0.71)	3.26 (±0.94)	4.16 (±0.57)	3.35 (±0.86)	2.14 (±0.93)
	Doctorate degree		2.93 (±0.74)	3.35 (±1.03)	4.22 (±0.57)	3.42 (±0.76)	2.03 (±0.89)
Your position	Administrative	Yes	2.71 (±0.74)	3.41 (±0.96)	4.39 (±0.37)	3.49 (±0.81)	1.94 (±0.93)
		No	2.97 (±0.74)	3.35 (±1.02)	4.19 (±0.59)	3.41 (±0.77)	2.08 (±0.90)
	Academic-researcher	Yes	2.93 (±0.74)*	3.33 (±1.02)	4.21 (±0.57)	3.39 (±0.77)**	2.03 (±0.89)**
		No	3.27 (±0.67)	3.68 (±0.90)	4.25 (±0.63)	3.85 (±0.67)	2.69 (±0.98)
	Research assistant	Yes	3.29 (±0.59)*	3.65 (±0.79)	4.21 (±0.60)	3.77 (±0.64)*	2.73 (±1.05)**
		No	2.92 (±0.75)	3.33 (±1.03)	4.21 (±0.57)	3.39 (±0.78)	2.01 (±0.87)
Institution type	Public university		3.01 (±0.76)*	3.32 (±1.06)	4.21 (±0.62)	3.37 (±0.77)	2.14 (±0.97)
	Private university		2.85 (±0.71)	3.39 (±0.95)	4.21 (±0.50)	3.51 (±0.78)	1.97 (±0.80)
Research Interest?	Medicinal chemistry	Yes	2.91 (±0.69)	3.59 (±0.82)**	4.26 (±0.55)	3.45 (±0.72)	2.17 (±0.89)*
		No	2.97 (±0.77)	3.20 (±1.09)	4.18 (±0.59)	3.40 (±0.82)	2.00 (±0.91)
	Biology	Yes	2.90 (±0.75)	3.61 (±1.03)**	4.27 (±0.55)	3.52 (±0.75)	2.38 (±0.95)**
		No	2.98 (±0.74)	3.19 (±0.97)	4.17 (±0.59)	3.36 (±0.78)	1.87 (±0.82)
	Medical fields	Yes	3.00 (±0.75)	3.50 (±0.96)**	4.29 (±0.57)**	3.45 (±0.77)	2.14 (±0.94)
		No	2.86 (±0.72)	3.12 (±1.06)	4.08 (±0.56)	3.38 (±0.77)	1.96 (±0.84)
Years of Research Experience	≤5		2.83 (±0.73)	3.36 (±0.89)	4.16 (±0.61)	3.19 (±0.81)**	1.93 (±0.84)**
	6-10		2.95 (±0.72)	3.44 (±0.98)	4.21 (±0.54)	3.46 (±0.77)	1.96 (±0.87)
	11-15		3.06 (±0.81)	3.51 (±0.98)	4.36 (±0.54)	3.59 (±0.78)	2.41 (±0.95)
	16-20		2.91 (±0.73)	3.39 (±1.2)	4.26 (±0.54)	3.50 (±0.66)	2.18 (±0.89)
	>20		3.12 (±0.72)	2.97 (±1.22)	4.12 (±0.59)	3.68 (±0.60)	2.14 (±0.99)

* *P* value ≤ .05.

** *P* value ≤ .01.

RR, readiness of the researcher; RSR, readiness of scientific research; ROVRC, respondents’ opinions regarding virology research components; RVR, readiness of virology research; SD, Standard deviation; WCVR, willingness for conducting virology research.

ANCOVA analysis revealed that the total readiness of virology research, including COVID-19 related research was associated with research interest and years of experience as shown in Table 4. In years of experience, having “less than 5 years of experience” was significantly lower than “11-15” y of experience group. Research interest in biology, medical field, and medicinal chemistry significantly increased the mean of total of virology research when compared with not having these research interests.

**Table 4:** Significant variables associated with total questionnaire mean (post hoc results)

Variable	Mean Difference	Standard Error	*P*-Value	95% Confidence Interval for Difference	
				Lower Bound	Upper Bound
“Less than 5 years’ experience” – “11-15”	−0.254	0.086	.003	−0.422	−0.850
“Biology” – “Not biology”	0.182	0.057	.002	0.070	0.295
“Medical field” – “Not medical field”	0.232	0.058	.000	0.118	0.346
“Medicinal chemistry” – “Not medical chemistry”	0.128	0.059	.030	0.013	0.243

The repeated measures ANOVA indicated that the score of ROVRC factor was significantly higher than the remaining factors (*P* < 0.01), and the scores of RR factor was significantly lower than the remaining factors (*P* < 0.01), it also indicated that there were significant differences between the scores of all the factors except between the scores of WCVR factor and RSR factor.

## Discussion

The COVID-19 pandemic is a public health emergency of international concern and poses a challenge to the research and scientific community.^25^ Research data are needed to develop evidence-driven decisions regarding the importance of establishing and activating human virology research during the epidemic crisis.^19^ Therefore, in this study, we designed and validated a questionnaire to measure the attitude of academics and researchers in the medical field and those whom their work may contribute to it toward the extent of readiness and effectiveness of the research concerning human viral infectious diseases, including COVID-19, in Jordan. The items of this questionnaire were designed based on the WHO requirements and guidelines on establishing of virology laboratory in developing countries. The items asked about the readiness and effectiveness of key aspects of establishing a virology laboratory in a developing country, policy and program, infrastructure, human resources, and technologies available.^3^ The overall response rate were considered to be acceptable as previously reported.^26^

The result of the EFA suggested a 5-factor model would fit the survey. These factors are RVR, WCVR, ROVRC, RSR, and RR. These factors were found to have acceptable factor loadings, communalities and high internal consistency. The total mean for these factors was also satisfactory with the highest total mean for ROVRC factor. After establishing construct validity, the finalized version of the survey consisted of 37 questions (9 questions for RVR, 7 questions for WCVR, 7 questions for ROVRC, 9 questions for RSR, and 5 questions for RR). Data show that factors ROVRC, RSR, and WCVR have the highest means while RVR and RR have the lowest. This indicates that academics and researchers in Jordan agree that the willingness of researchers, and readiness of scientific research are available and effective in Jordan. Also, the respondents’ opinions agreed on the importance of the availability of the components for virology research, including COVID-19 related research. However, we lack the readiness component of both the researchers and research. This is because Jordan needs better infrastructure to accommodate specifications required for virology research as well as better training programs and competent researchers to conduct this type of research.

The result of the survey indicated that the position in the institution was significantly associated with RVR, RSR, and RR. Academic researchers revealed that, in Jordan, we are not ready for conducting virology or scientific research. This might be due to the limited resources for research in Jordan. At the time of the study, no official grants were allocated toward research concerning viruses. Furthermore, virology research was not considered as 1 of the national priorities. Importantly, bachelor degree holders were more ready to conduct research than other educational levels. This might be due to the fact that bachelor degree holders had not yet encountered the obstacles presented by limited resources. While higher education levels have faced these limitations, which makes them reluctant to engage in research especially virological research without the suitable resources. Public universities have higher readiness for virology research. That might be due to the availability of resources and that public universities focus more on research. Research interests in medicinal chemistry and biology were more ready and willing to conduct research, especially virology-related research. This is not surprising as the main focus of research is in finding better and new drugs that to help humanity in different disciplines. Since the COVID-19 outbreak, the focus of research has shifted toward virology-related research. Finally, the years of experience have also influenced the readiness and the willingness to conduct research, including virology research. The more years of experience, the more ready to be a researcher. That is also unsurprising as more experience will make it easier to overcome obstacles encountered during research and the faster the research is conducted.

According to Guidelines on Establishment of Virology Laboratory in Developing Countries prepared by WHO, Jordan lacks the requirements to establish a virology research laboratory related to COVID-19. Knowing the effects of SARS-CoV-2 and based on the WHO guidelines, it is related to Risk Group 4 that possess “high individual and community risk.” Hence, dealing with SARS-CoV-2 requires the following: a laboratory type of “dangerous pathogens unit,” a “Level 3 plus airlock entry, shower exit, special waste disposal,” and “Class III biological safety cabinet (BSC), or positive pressure suits in conjunction with Class II BSCs, double-ended autoclave (through the wall), filtered air.” Unfortunately, Jordan lacks these requirements.

Furthermore, based on the aforementioned guideline, minimum staff requirements are of great importance. These include “A qualified virologist possessing a postgraduate qualification in virology with 3 to 5 years’ experience in diagnostic virology,” “Two junior microbiologists possessing a Master’s degree in Medical Microbiology with 1 to 2 years’ experience in diagnostic virology,” “Two laboratory technologists possessing a graduate degree in science with a diploma in Medical Laboratory Technology (1 to be trained in cell culture and virus isolation methods and the other to be trained in serology),” and “One or two laboratory supportive staff.” Although Jordan might have the previous qualifications in terms of degrees obtained internationally through postgraduate studies, but they definitely will not have the experience in diagnostic virology as we already lacking the proper facilities.

During a public health crisis in which viruses are involved, it is essential for policy-makers and public health experts to know the extent of readiness of human virology research. Having access to data on virology research components such as the number of qualified academics and researchers who are interested in studying human viral infectious diseases, the presence and readiness of diagnostic and research virology laboratories, the presence of proper infrastructure, the allocation of institutions’ budget intended for establishing virology laboratory and for supporting the human virology projects, and the presence of policies and procedures to conduct human virology research allows evidence-based decisions, including what components to focus on when developing human virology research to facilitate the diagnosis and discovery of vaccines and other treatments.^27^

Laboratories have complex structures and are diverse in purpose.^28^ Today’s biomedical research and clinical laboratories must have a dynamic nature and be able to adapt rapidly to public health pressures and needs.^29^ Indeed, emerging and/or re-emerging of infectious diseases is 1 example where laboratories must be able to adjust priorities to meet with the challenges facing them. Thus, regular certification should be conducted for all biological research and clinical laboratories to guarantee that adaptation and maintenance are undertaken promptly and in an appropriate and safe manner.^30^ Similarly, to allow virology research to be conducted promptly, all necessarily research components including human and physical resources, funds, and policies should be available to the researchers. Establishing a national virology laboratory would allow local scientists to quickly sequence the new virus gene and study an outbreak rather sending the samples of diseases and viruses to laboratories abroad. By doing so, time is saved and, consequently, so are lives.^31^ We need scientists and researchers from a wide variety of disciplines to work together to stop the COVID-19 outbreak and to prepare for future outbreaks.

We found that the RR factor was associated with several factors, including gender, education level and researchers’ interest. We need scientists and researchers from a wide variety of disciplines to work together to stop outbreaks, but this will not occur unless we increase the readiness of researchers and research concerning virology in Jordan. Thus, we recommend establishing a virology laboratory that possesses the recommended infrastructure, as well as to create a training program that trains to increase researchers’ readiness to conduct virology research, such as COVID-19 research.

It is worth mentioning that there are number of academic and research institutions in Jordan that will be funding or are currently funding COVID-19 research projects. For example, the Abdul Hameed Shoman Foundation has started to receive research proposals and Al-Zaytoonah University of Jordan funded 2 research projects. However, it is known that these research projects will not be dealing with the virus itself, because we lack properly trained researchers and proper research in virology. These projects are likely to be dealing with coronavirus from different research aspects, such as informatics.

## Future Work

This article represents the first step of a long-term and more comprehensive validation research to ensure that the virology research is optimized. Future work may include implementing this survey on other countries. This will help in uncovering the difficulties and obstacles that may hinder virology research. Moreover, feedback from this survey will help improve and enhance virology research to respond to future pandemics.

## Limitations

Several limitations could be discussed for this study. The participants, who completed the questionnaire, could be more interested in the topic than those who did not complete it, which may lead to selection bias. Furthermore, recall bias may have affected recollections of events or interactions by participants. Likewise, pre-existing ideas can also impair the memory of past events and their responses were not independently validated. As the questionnaire was completed by the participants, social desirability bias could be present. However, the identities of the respondents were anonymous.

In conclusion, due to the increase of the demand for virology research globally during COVID-19 pandemic crisis, it was necessary to measure the level of readiness and effectiveness of virology research in Jordan. Therefore, we developed and tested the content reliability and content validity of a questionnaire-based evaluation tool designed for evaluating the readiness and effectiveness of virology research in Jordan. Data showed that researchers are willing to conduct virology research due to readiness of the researchers in scientific research. However, in Jordan, we lack the readiness of both the researchers and research in virology to do this. Authorities should pay attention for these shortcomings in virology research with the help of academia.

## Supplementary material

For supplementary material accompanying this paper visit http://dx.doi.org/10.1017/dmp.2020.437. click here to view supplementary material
