# Supplementary material for: Conducting COVID-19-Related Research in Jordan: Are We Ready?
Source: Disaster Med Public Health Prep. 2020 Nov 5:1–8. doi: 10.1017/dmp.2020.437 (PMC8010288; doi:10.1017/dmp.2020.437)
Supplement: Supplementary file 1 [file S1935789320004371sup001.docx]

**Readiness and effectiveness of the research process involving the study of human viral infectious diseases**

Dear Researcher:

Amid the spread of the global pandemic COVID-19 caused by the emerging Coronavirus, we ask you to fill the following questionnaire. This questionnaire aims to assess:

1. Your opinions about the readiness of biosafety facilities and human resources to conduct laboratory-based research in the field of human viral infectious disease including, COVID-19.
2. Your willingness to conduct laboratory-based research in the field of human viral infectious diseases including, COVID-19.
3. Your satisfaction with the policies concerning the conduction of a research in the field of human viral infectious diseases including, COVID-19.

**Gender:**

- Male
- Female

**Age:…………………………**

**Education level:**

- Bachelor's degree.
- Master's degree.
- Doctorate degree.

**General Specialization:**

- Medicine
- Veterinary
- Dentistry
- Pharmacy Doctor
- Pharmacy
- Nursing
- Medical Laboratory Science
- Genetic Engineering
- Chemistry
- Biology
- Other, please mention your EXACT specialisation ---------------------

**Years of experience in scientific research (after the highest academic degree obtained):**

- <1
- 1-5
- 6-10
- 11-15
- 16-20
- >20

**Please select your institution (you can select more than one):**

- Public University
- Private University
- Research Centre (independent or within a university)
- Infectious Diseases Research Centre

**Your institution's city**

**…………………………..**

**Your position in the institution (you can select more than one):**

- Teaching assistant
- Research assistant
- Technician
- Researcher
- Academic
- Administrative

**Do you have a research interest in any of the following scientific fields (you can select more than one)?**

- Drug Design
- Drug Discovery
- Drug Screening
- Drug Synthesis
- Drug Formulation
- Drug Kinetics
- Target Discovery
- Epidemic Research
- Pandemic Research
- Clinical Care
- Bioengineering
- Informatics/Bioinformatics
- Biochemistry
- Pathology
- Pharmacology
- Microbiology
- Public Health
- I do not have a research interest
- Others, please mention your research interest…………………

1. I have a previous or current laboratory-based research concerning the study of human viral infectious diseases (including COVID-19)
   - Strongly disagree
   - Disagree
   - Neutral
   - Agree
   - Strongly agree
2. I have experience and/or qualification(s) to conduct laboratory-based research in the field of human viral infectious diseases (including COVID-19)
   - Strongly disagree
   - Disagree
   - Neutral
   - Agree
   - Strongly agree
3. I have received sufficient funding from MY institution to conduct a research in the field of human viral infectious diseases (including COVID-19)
   - Strongly disagree
   - Disagree
   - Neutral
   - Agree
   - Strongly agree
4. I have received sufficient funding from OTHER institutions (national or international) to conduct a research in the field of human viral infectious diseases (including COVID-19)
   - Strongly disagree
   - Disagree
   - Neutral
   - Agree
   - Strongly agree
5. I have a joint laboratory-based research collaboration with national and/or international institutions (including the healthcare sector) to conduct a research in the field of human viral infectious diseases (including COVID-19)
   - Strongly disagree
   - Disagree
   - Neutral
   - Agree
   - Strongly agree
6. I am interested in attending or participating in national and/or international scientific symposia concerned with the latest developments in the field of human viral infectious diseases (including COVID-19)
   - Strongly disagree
   - Disagree
   - Neutral
   - Agree
   - Strongly agree
7. I am willing to submit a proposal for laboratory-based research in the field of human viral infectious diseases (including COVID-19)
   - Strongly disagree
   - Disagree
   - Neutral
   - Agree
   - Strongly agree
8. I am willing to help in establishing a standard diagnostic and research virology laboratory (including COVID-19 laboratory)
   - Strongly disagree
   - Disagree
   - Neutral
   - Agree
   - Strongly agree
9. I am willing to participate in training courses needed for the conduction of laboratory-based research involving human viral infectious diseases (particularly for COVID-19)
   - Strongly disagree
   - Disagree
   - Neutral
   - Agree
   - Strongly agree
10. I am willing to collaborate with national and/or international institutions (including the healthcare sector) to conduct laboratory-based research in various fields of human viral infectious diseases (including COVID-19)
    - Strongly disagree
    - Disagree
    - Neutral
    - Agree
    - Strongly agree
11. Provided the availability of biosafety facilities, I will try to link my current project with the research involving human viral infectious diseases (including COVID-19)
    - Strongly disagree
    - Disagree
    - Neutral
    - Agree
    - Strongly agree
12. Provided the availability of biosafety facilities, I am willing to do educational research training in the field of human viral infectious diseases (particularly for COVID-19)
    - Strongly disagree
    - Disagree
    - Neutral
    - Agree
    - Strongly agree
13. My institution has well-equipped research laboratories
    - Strongly disagree
    - Disagree
    - Neutral
    - Agree
    - Strongly agree
14. My institution has multidisciplinary research laboratories
    - Strongly disagree
    - Disagree
    - Neutral
    - Agree
    - Strongly agree
15. My institution has standard diagnostic and research virology laboratories (including COVID-19 laboratory)
    - Strongly disagree
    - Disagree
    - Neutral
    - Agree
    - Strongly agree
16. Regular maintenance of tools and equipment is available in my institution research laboratories
    - Strongly disagree
    - Disagree
    - Neutral
    - Agree
    - Strongly agree
17. Safety measures are employed efficiently in my institution research laboratories
    - Strongly disagree
    - Disagree
    - Neutral
    - Agree
    - Strongly agree
18. In my opinion, there are urgent needs to establish national diagnostic and research virology laboratories and to financially support human viral infectious diseases projects (including COVID-19 projects)
    - Strongly disagree
    - Disagree
    - Neutral
    - Agree
    - Strongly agree
19. In my opinion, there is a high need to collaborate with international virology laboratories to benefit from their experience and knowledge
    - Strongly disagree
    - Disagree
    - Neutral
    - Agree
    - Strongly agree
20. My institution follows biosafety guidelines for the management of general laboratory waste, including biological hazards (if available)
    - Strongly disagree
    - Disagree
    - Neutral
    - Agree
    - Strongly agree
21. In my opinion, biosafety guidelines should be created and implemented for the prevention of and dealing with biological hazards (including viral hazard)
    - Strongly disagree
    - Disagree
    - Neutral
    - Agree
    - Strongly agree
22. My institution provides financial support for research projects in various fields
    - Strongly disagree
    - Disagree
    - Neutral
    - Agree
    - Strongly agree
23. Part of my institution's budget is allocated for establishing well-equipped research laboratories
    - Strongly disagree
    - Disagree
    - Neutral
    - agree
    - Strongly agree
24. The infrastructure of my institution allows the establishment of multidisciplinary research laboratories, including diagnostic and research virology laboratory (such as COVID-19 laboratory)
    - Strongly disagree
    - Disagree
    - Neutral
    - Agree
    - Strongly agree
25. Part of my institution's budget is allocated for establishing standard diagnostic and research virology laboratories (including COVID-19 laboratory)
    - Strongly disagree
    - Disagree
    - Neutral
    - Agree
    - Strongly agree
26. My institution provides financial support for participating in national and/or international training courses in various fields including virology training courses (such as COVID-19 courses)
    - Strongly disagree
    - Disagree
    - Neutral
    - Agree
    - Strongly agree
27. In my opinion, there is an urgent need for my institution to financially support the participation in training courses involved in various field of virology (including COVID-19)
    - Strongly disagree
    - Disagree
    - Neutral
    - Agree
    - Strongly agree
28. My institution financially supports national and/or international joint research projects that are involved in the study of human viral infectious diseases (including COVID-19)
    - Strongly disagree
    - Disagree
    - Neutral
    - Agree
    - Strongly agree
29. In my institution, there are reasonable numbers of employees who are/were conducting research in various fields of human viral infectious diseases
    - Strongly disagree
    - Disagree
    - Neutral
    - Agree
    - Strongly agree
30. In my opinion, there is a high need to increase the number of microbiologists (including virologist) in my institution to enable the conduction of a research in the field of human viral infectious diseases (including COVID-19)
    - Strongly disagree
    - Disagree
    - Neutral
    - Agree
    - Strongly agree
31. My institution has a permit from governmental agencies and/or the Deanship of Scientific Research to conduct laboratory-based research in the field of human viral infectious diseases (including COVID-19)
    - Strongly disagree
    - Disagree
    - Neutral
    - Agree
    - Strongly agree
32. My institution has strict policies regarding laboratory-based research procedures involving human viral infectious diseases (including COVID-19)
    - Strongly disagree
    - Disagree
    - Neutral
    - Agree
    - Strongly agree
33. My institution has research monitoring committees that audit and follow up on the proposed and current human viral infectious diseases projects (including COVID-19 projects)
    - Strongly disagree
    - Disagree
    - Neutral
    - Agree
    - Strongly agree
34. My institution has strict policies regarding researchers' compliance with standard procedures and safety guidelines when conducting human viral infectious diseases project (including COVID-19 projects)
    - Strongly disagree
    - Disagree
    - Neutral
    - Agree
    - Strongly agree
35. My institution has clear regulations that allow joint research collaborations with various institutions (including the health care sector)
    - Strongly disagree
    - Disagree
    - Neutral
    - Agree
    - Strongly agree
36. In my opinion, there is an urgent need to create governmental policies for controlling research process concerned with human viral infectious diseases (including COVID-19)
    - Strongly disagree
    - Disagree
    - Neutral
    - Agree
    - Strongly agree
37. In my opinion, there is an urgent need to create institutional policies for controlling research process concerned with human viral infectious diseases (including COVID-19)
    - Strongly disagree
    - Disagree
    - Neutral
    - Agree
    - Strongly agree
